# Symptoms of selective mutism beyond failure to speak in children and adolescents

**DOI:** 10.1007/s00787-024-02415-9

**Published:** 2024-03-27

**Authors:** Felix Vogel, Carolin Röse, Christina Schwenck

**Affiliations:** 1 Department of Child and Adolescent Psychotherapy, University of Hamburg, Hamburg, Germany; 2 Vitos Children and Adolescent Clinic for Mental Health Marburg, Hamburg, Germany; 3 Department of Clinical Child and Adolescent Psychology, University of Giessen, Giessen, Germany

**Keywords:** Selective mutism, Anxiety disorder, Symptomatology, Qualitative content analysis, Diagnostic criteria, Children

## Abstract

Understanding the symptoms of a mental disorder is essential for accurate diagnosis or selecting appropriate treatment targets. Despite this, there is a surprising lack of systematic research on the symptoms of selective mutism (SM). While the DSM-5 defines failure to speak as the only core symptom of SM, sparse research suggests that children with SM may experience additional symptoms. Previous studies have been limited in their identification of symptoms of SM, either by using a predefined set of symptoms or by only asking for anxiety-specific symptoms. This may have resulted in important symptoms being overlooked. In this study, we provided *n* = 86 parents of children and adolescents with SM (3–18 years) with a symptom definition appropriate for the target group. Additionally, parents were asked an open-ended question about any other symptoms they had observed in their children, beyond the failure to speak. The symptoms reported were categorized using qualitative content analysis (QCA) and examined for frequency and association with symptom severity. Ten different symptom categories were identified, with fear, freezing, and avoidance/security behaviors being the most prevalent. On average, parents reported *M* = 4.74 (SD = 2.37) symptoms from different symptom categories. Only fear was found to be related to symptom severity of SM. As the findings suggest that SM encompasses various symptoms beyond failure to speak, a more sophisticated understanding of SM as a mental disorder with multiple symptoms seems essential. The clinical implications of this are discussed in further detail.

## Introduction

Selective mutism (SM) is characterized by a consistent failure to speak in certain social situations, despite being able to speak in other situations [1]. To diagnose SM, DSM-5 defines additional diagnostic criteria (e.g. impairment, presence of the disorder for at least one month) in addition to the core symptom of failure to speak [1]. The Diagnostic Features and Associated Features to Support the Diagnosis of SM sections of SM in DSM-5 include additional clinical features such as high levels of social anxiety, withdrawal, externalizing behaviors, and impairments in communication skills [1]. These clinical features reflect the findings that (social) anxiety is a central phenomenon in the majority of children with SM [2, 3], and that also additional symptoms beyond anxiety (e.g. externalizing behavior, delayed speech development) may occur in affected children [4]. SM causes severe impairments in academic and social functioning [5, 6], typically emerges during preschool age [7] and can last for several years [8]. According to epidemiological studies, the average prevalence is 1% [3, 7].

Despite the severe impairments, chronic course, and high incidence of unreported cases, it is surprising that the diagnostic criteria of SM in DSM-5 do not meet the definition of a mental disorder. According to DSM-5, mental disorders are broadly defined as a syndrome (a multitude of covarying symptoms) resulting in clinically significant emotional, cognitive, and/or behavioral impairment ( [1], p. 20). While it is acknowledged that SM can result in impairments [5, 6], the diagnostic criteria for SM in DSM-5 only define a single symptom, namely failure to speak, without specifying a syndrome. Considering the high importance of anxiety in SM [3] and first evidence of additional symptoms in children with SM (see below), the diagnostic criteria of SM may be imprecise. The precision of diagnostic criteria is crucial because a valid diagnosis based on empirically identified symptoms is necessary to identify individuals affected by a specific mental disorder, to apply a particular treatment approach, or to study a mental disorder’s pathomechanisms [9]. Knowing which pathognomonic symptoms occur in a clinical group therefore has far-reaching implications for research and clinical practice and emphasizes the importance of systematic research on symptoms.

The term symptom in the context of psychopathology is not consistently defined [10, 11]. Following the theory-overarching symptom definition of Wilshire et al. [10], we define psychopathological symptom as a persistent or recurrent behavior or subjective experience (cognition, emotion, or bodily sensation), which is distressing and/or impairs function, is considered to be indicative of an underlying mental problem and can be self-observable and/or observable by a third person [10]. So far, two studies directly asked parents of children with SM about symptoms in their children using a standardized list of possible clinical features [8, 12]. In the study of Remschmidt et al. [8] the symptoms (in addition to the absence of speech) lack of contact (76% of individuals), psychomotor disturbances (76%), low self-esteem (78%) and pronounced anxiety (80%) stood out clearly in their frequency from the other symptoms asked about. However, the authors did not provide a description of the symptoms. Therefore, it is unclear which features are included in pronounced anxiety or what exactly is meant by lack of contact or psychomotor disturbances. In the study of Ford et al. externalizing symptoms (e.g. temper tantrums, disobedient at school), anxiety-related symptoms (e.g. fearful, clings to adults) and regressive behavior were among the top-ranked symptoms [12]. However, it is important to note that symptoms were only considered to be present on the basis of CBCL items as 'sometimes', 'often' or 'very often'. This means that occasional and developmentally typical symptoms, such as occasional temper tantrums, may be mistakenly seen as pathological. The most commonly reported symptoms were externalizing symptoms (e.g. temper tantrums, disobedient at school), anxiety-related symptoms (e.g. fearful, clinging to adults) and regressive behavior. In a third study, children and adolescents with SM were asked about circumscribed fears that caused them to remain silent. The answers were provided in an open-ended format [2]. This study demonstrates that circumscribed fears are prevalent in a majority of children and adolescents with SM, using a qualitative approach that does not restrict possible symptoms to a predetermined list. However, the authors only assessed the emotional dimension of fear, and therefore individuals did not report any possible symptoms unrelated to fear or behavioral anxiety-related symptoms. Moreover, other studies have used various methods to assess symptoms in SM related to anxiety indirectly (e.g. sum scores of questionnaires or physiological reactions) [e.g. 13, 14], which are therefore not suitable for identifying specific symptoms.

Taken together, it appears that previous research is beginning to provide evidence of symptoms of SM beyond failure to speak. However, studies have only focused on anxiety-specific symptoms or a predefined set of symptoms, potentially leaving important symptoms unidentified. Therefore, a systematic study that does not restrict the assessment to a predefined set of symptoms or limit it to certain domains (such as emotions or fear) is needed. Therefore, the present study aims to investigate whether additional psychopathological symptoms beyond the failure to speak occur in children and adolescents with SM and if so, which ones. For this purpose, parents were asked to complete an online survey and provide information on the symptoms their children experience. The question about symptoms was in an open response format and included a definition of symptoms appropriate for the target audience. Given that a wide range of possible symptoms has never been systematically investigated in the context of SM using an open response format, this study will be mainly exploratory in nature. However, based on previous research [2, 8, 12] and symptoms listed in the DSM-5 for other anxiety disorders in children [1], we expect to observe typical behavioral fear responses being reported (feeling of fear, avoidance, freezing, tantrums, clinging, whining), as well as symptoms related to externalizing behavior.

## Materials and methods

### Sample

Initially, *n* = 262 parents started the online study, of whom *n* = 91 completed the questionnaire. Inclusion criteria comprised an age of children and adolescents between 3 and 17 years and exceeding the cut-off value on the diagnostic instrument for SM. A number of *n* = 5 did not exceed the cut-off value, resulting in a final sample of *n* = 86 individuals.

### Procedure

Parents of children with SM were recruited from various sources throughout Germany, including online forums, letters to inpatient and outpatient clinics. All parents completed an online questionnaire using the Unipark survey platform. Initially, parents were informed about the study and informed consent was obtained by button press. An educational video was presented to illustrate the concept of symptoms (see materials). Subsequently, we asked parents to provide an open response about symptoms beyond failure to speak experienced by children and adolescents with SM in situations where speaking is expected. Afterwards, parents conducted the Frankfurt Scale of Selective Mutism to assess SM. The open question about symptoms was placed at the beginning of the questionnaire to minimize the influence of diagnostic questionnaires on the open responses. The study received approval from the Ethics Committee of the Faculty of Psychology and Sports Science at the University of Giessen (Germany).

### Materials

#### Frankfurt scale of selective mutism (FSSM)

The FSSM is a parent-rated screening questionnaire for SM in children aged 3 and 18 years [15] and includes a diagnostic scale (DS) and a severity scale (SS). The authors report excellent reliability (*α* = 0.90–0.98) and validity based on a one-factor solution of the SS. Reliability was also excellent in the present sample (*α* = 0.92–0.93). In the study at hand, recommended cut-off values of DS [15] were used to identify clinically relevant SM. Relative scores of the SS were applied (range 0–1).

#### Diagnostic system for mental disorders according to ICD-10 and DSM-5 for children and adolescents, parent report for social anxiety disorder (DISYPS-III, FBB-SOZ)

The FBB-SOZ of the DISYPS-III (referred to as DISYPS-SOZ) [16] is a parent rating scale with seven items on symptoms of social anxiety disorder. Items are rated on a four-point Likert scale (0 = not at all true, 3 = completely true), and internal consistency (*α* = 0.736) was satisfactory in the present sample.

#### Educational video and open-ended question

The video was based on Wilshire et al. [10] symptom definition and used drawn animations from videoscribe software to provide examples for symptoms in three contexts: (a) physical condition (e.g., pain as a symptom of a broken arm), (b) externalizing psychopathology (e.g., being in a conflict with another peer), (c) internalizing psychopathology (e.g., fear-related thoughts when confronting a spider). The presentation covers a broad spectrum of possible symptoms. After watching the video, parents were requested to report their child's feelings, thoughts, senses, or actions in a typical situation where the child is expected to speak but fails to do so. Parents were instructed to write each symptom in separate text boxes (maximum 255 characters) within the online survey, allowing a maximum of 10 symptoms.

### Data analysis

A qualitative content analysis (QCA) was conducted to categorize responses into symptom categories for each situation. QCA is a widely accepted method to analyze qualitative data such as open-ended reports from patients. We applied QCA according to recommendations in the literature [17], and the process used in the previous SM study on fears was mirrored (see for further details [2]). Two approaches were used to calculate symptom frequencies. Firstly, the proportion (%) of individuals per category reporting a symptom was determined. Secondly, the proportion (%) of a symptom category reported relative to all symptoms was calculated. Interrater reliability was tested by three independent, blinded researchers who assigned responses to higher-order categories. Prototypical examples for each category ensure trustworthiness [17]. We calculated correlations between symptom categories from QCA and child characteristics (age, gender, SM severity, social anxiety). Point biserial correlations were used to assess the association between symptom categories (coded with 0/1) and age and symptom severities, while Phi coefficients were used to measure the correlation between symptom categories and gender. The analyses were conducted using IBM SPSS 28 and Rstudio’s psych-package [18].

## Results

### Sample characteristics

Out of the total *n* = 86 cases, the online questionnaire was completed by the biological mother in *n* = 84 cases (97.7%) and by the biological father in *n* = 2 cases (2.3%). The study included children and adolescents aged between three and 17 years (*M* = 9.67, SD = 4.37; 66% female), with only five children and adolescents (5.8%) growing up multilingually. In 5.8% of families, one parent, and in 3.5% of families, both parents were outside Germany. German was spoken at home in *n* = 81 participating families (94.2%). Of the children, 26.7% attended kindergarten, 29.1% attended elementary school, 20.9% attended secondary school, and 15.1% attended another type of school such as a vocational school. The mean sum score of the DS was *M* = 9.09 (SD = 0.97; range: 0–10, cut-off = 6 or 7), and the mean relative score of SS was *M* = 0.65 (SD = 0.12; range: 0–1).

### Open-ended questions

#### Reported symptoms and extracted symptom categories

**Fig. 1:**
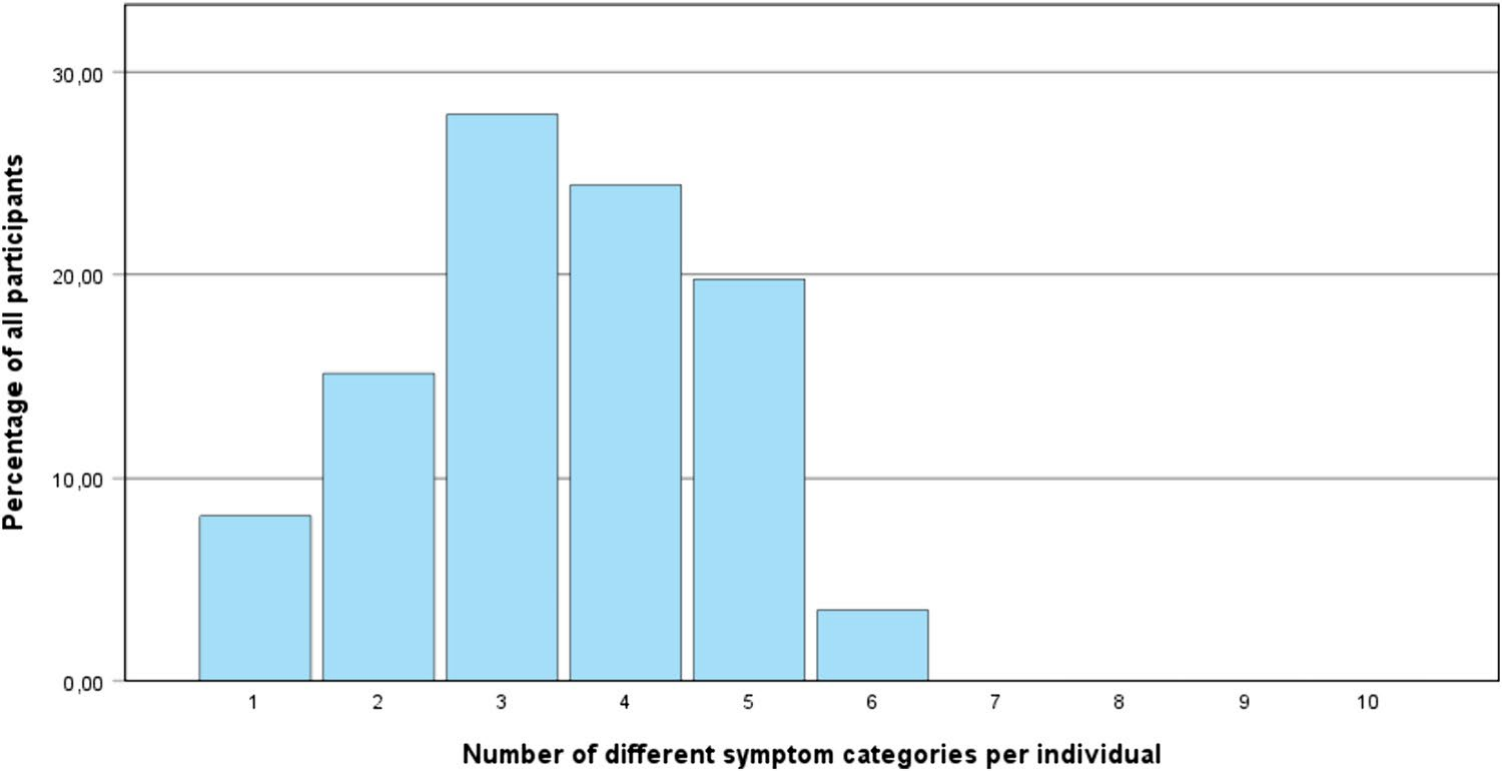
Percentage of individuals per number of reported symptoms from different symptom categories per individual. Because ten different symptom categories were identified in the present study, a maximum of symptoms from ten different categories could be reported

Overall, 98.8% (n = 85) of individuals provided at least one codable symptom for extracting and assigning categories. This resulted in 427 codable symptoms across all individuals, with an average of *M* = 4.74 (SD = 2.37) symptoms per individual (range: 1 to 6 symptoms of different categories beyond failure to speak, see Fig. 1). Out of the total responses received, 43 responses were deemed uncodable due to various reasons such as absence of a symptom ("in unknown situations"), reporting of the presupposed symptom failure to speak ("it becomes silent”), unspecific descriptions ("speech barrier"), or lack of knowledge ("I don’t know”). Therefore, 88.6% (427 out of 482) of the responses were identified as symptoms categorized accordingly. The number of codable responses was not affected by sample characteristics such as symptom severity, age, or gender. We extracted ten higher-order categories of symptoms using QCA from the 427 codable symptoms (Table 1). Both interrater reliability agreements between the first-rater (first author) and the second-rater (last author) as well as the first rater and third-rater (second author) can be considered strong [2], indicated by Kappa coefficients of *κ* = 0.86 and *κ* = 0.80 respectively. Table 1 shows the extracted symptom categories, exact criteria for assigning symptoms, frequencies, and prototypical examples.

**Table 1:** Categories of reported symptoms with descriptions and frequencies

Symptom category	Description	% of parents who reported a symptom of this category	% of reported symptoms compared to all reported symptoms	Prototypical examples
Fear	A feeling of fear including non-specific descriptions of fears, uncertainty, panic, description of fears with circumscribed content or physiological reactions related to fear	66.3%	22%	“Marked fear” “Fear of making a mistake” “Breathing becomes faster” "Blushing” “stomach ache”
Freezing	A state of immobility of the entire body or individual body parts including facial expressions and eye movements, a physical tension, cramping, or increased muscle tone, slowed or inhibition of motor function or reduced responsiveness as well as unresponsiveness	65.1%	22%	“The face freezes, all facial expressions are frozen” “Body tension is very strong and seems petrified and frozen” “He stands still and freezes completely” “She is not responsive”
Avoidance / safety behavior	Behaviors aiming at leaving or refusing certain situations, avoiding to get into a certain situation, behaviors that serve to calm in threatening situations, careful preparation in advance of a situation or averting gaze from social counterpart by looking away or turning away	61.6%	22%	“Leaves the situation suddenly” “Avoids people who might speak to him” “Laughs at unintelligible questions” “Go through upcoming procedures very carefully and ask for details” “Avoids eye contact”
Self-esteem impairment / negative affect	Negatively connoted feelings, reduced self-worth, negative thoughts about oneself or dissatisfaction with oneself or one's abilities	27.9%	11%	“Feels helpless”, “Lack of self-esteem” “He is extremely disappointed about himself because he always thinks he is failing”
Symptom category	Description	% of parents who reported a symptom of this category	% of reported symptoms compared to all reported symptoms	Prototypical examples
Externalizing behavior	Aggressive behavior against other people or objects as well as anger and temper tantrums	26.7%	7%	“Boxes or beats us parents”, “massive uncontrolled outbursts of anger”
Reassuring behavior	Child's initiation of contact with the parents/ a caregiver through gestures, facial expressions, clinging behavior, or verbal communication or behavior that describe that the child communicates via another person	18.6%	5%	“Tries to send signals/calls for help to familiar person without words” “Help-seeking look to parents”, “clinging to our body” “Calling for mom” “Sends the younger brother ahead to do the talking”
Regressive behavior or displacement activity	Regressive behaviors and behaviors or movements that appear to be inappropriate or nonpurposeful for the situation	18.6%	4%	“Can no longer do things that she has mastered such as dressing independently, brushing teeth, making bread” “makes animal sounds such as barking”; “puts finger in mouth”
Whining	Whiny behavior or whining	15.1%	4%	“He weeps”
Reduced communication	Decreased, toneless or quiet speech, use of gestures to communicate, or inability to talk about one's feelings and thoughts	10.5%	3%	“Whispering” “Opens his mouth, his open mouth moves” “As an answer may come maybe a nod” “She does not talk about how she feels”
Reduced body tension and slackness	Loosening of body tension, either of the entire body or of individual body parts	4.7%	1%	“Posture slackens” “His face slackens” “Reduced tension of his whole body”

Symptom categories based on reports of *n* = 86 parents of individuals with SM, descriptions of the symptom categories and percentages with which the symptom category occurred among all individuals and with which the symptom category occurred in relation to all reported symptoms

### Correlational analyses

#### Correlations between symptom categories and child characteristics

There was no significant relationship found between dichotomous symptom categories and the child's age (*p* = 0.845). However, the symptom category of fear was significantly negatively correlated with gender (*r* = 0.27, *p* = 0.02), indicating that this symptom occurs less frequently in boys. Correlational analysis showed that a greater frequency of the symptom category fear was associated with higher SM severity (FSSM-SS) and higher expression of social anxiety in children (DISYPS-SOZ) (SM severity: *r* = 0.28, *p* = 0.01; social anxiety: *r* = 0.38, *p* < 0.001). Additionally, the child's level of social anxiety (DISYPS-SOZ) was also associated with a lower frequency of the symptom category whining (*r* = −0.24, *p* = 0.03).

## Discussion

The present study aimed to examine psychopathological symptoms beyond failure to speak in children and adolescents with SM. To the best of our knowledge, we provided the first empirical outline of possible symptoms of SM beyond failure to speak without using a predefined list or prior theoretical restrictions (e.g. anxiety). Our results replicated findings from previous studies and add symptoms not systematically described in SM before.

### Do children and adolescents with SM experience additional symptoms beyond failure to speak?

Our results suggest that children and adolescents with SM experience multiple symptoms, with an average of *M* = 4.74 symptoms from different categories. This aligns with previous studies [2, 8, 12], indicating additional symptoms beyond failure to speak in SM. As no parent reported more than six different symptom categories, symptoms of SM, at least those symptoms observable by parents, seem to be limited to a maximum of six different symptoms per individual. Therefore, it is unlikely for any child or adolescent with SM to exhibit all potential symptoms. This aligns with findings from other mental disorders, in which individuals often share core symptoms but vary in additional symptoms [16, 19]. The symptoms' heterogeneity may be based on various subtypes with distinct pathomechanisms [19]. While specific symptom profiles couldn't be drawn from our results, the study may have outlined potential symptoms that potentially occur in symptom profiles of SM and thus might provide a good starting point for identification of subtypes and pathomechanims.

### Which psychopathological symptoms are present in SM?

As expected, our findings suggest that children and adolescents with SM show predominantly anxiety-related symptoms such as fear, freezing or avoidance, but also symptoms, albeit less prominently, that may not be primarily driven by anxiety, such as impaired self-esteem and externalising behaviors. This essentially reflects that anxiety is the central phenomenon of most children with SM as indicated by the higher order category of anxiety disorders in DSM-5 and epidemiological research [3, 20]. Furthermore, the findings suggest that additional clinical features beyond anxiety are relevant in SM, supporting the view of SM as a heterogeneous disorder. [4].

#### Anxiety-related symptoms

As expected, we found that anxiety-related symptoms, such as fear, freezing, and avoidance/safety behavior, were the most commonly reported among individuals with SM. This supports the classification of SM as an anxiety disorder [1] and is consistent with prior research on symptoms of SM [2, 8, 12]. However, this current study builds upon prior research while addressing their methodological limitations in identifying unknown symptoms and their relevance. Previous studies relied on a predetermined list of symptoms [8, 12], failed to define reported symptom categories [8], included potentially normative behaviors as symptoms [12] or limited reported symptoms to the domain of anxiety [2]. For example, previous studies may have included symptom categories that obscure anxiety-related symptoms. Reduced eye contact, which is a safety behavior, may have been classified as 'lack of contact' (e.g. 76% of individuals behavior [8]), and motor inhibition as part of a freezing response may have been assigned to 'psychomotor disturbances' (e.g. 76% of individuals [8]). Therefore, the current study provides a more precise representation of these vague symptom categories from prior research. Additionally, current findings indicate that marked fear is a central phenomenon among most individuals with SM, making it a potential core symptom of the disorder. It is worth noting that we did not specifically inquire about fear in our study, unlike the previous study that employed an open-response format [2]. Nevertheless, our findings demonstrate that typical fear responses outlined in DSM-5 for childhood (such as avoidance, freezing, clinging, whining, and tantrums) [1] are also present in individuals with SM. While freezing may be a relevant pathomechanism in SM [13][13], and avoidance/safety behaviors are transdiagnostically relevant to anxiety disorders [21], the fear responses of clinging, crying, tantrum have been less studied in children with anxiety disorders (e.g., [22]. Therefore, the conclusion that the behavior described here occurs due to a fear response is not well-founded and requires further research. Moreover, the observed symptoms of the regressive behavior/displacement activity category may also be partially interpreted as a fear response. According to biological models of fear, displacement activity is an inappropriate action resulting from a conflict between two incompatible actions, such as avoidance and approach [23, 24]. Regressive behaviors may also be explained in the context of developmental delays, which occur in children with SM [3]. However, this is a new hypothesis in the SM literature that situationally inappropriate behavior in SM may also result from an fear response, which may have important implications for making diagnoses of SM in distinction to developmental delay.

The symptom category of reduced communication, specifically speaking at a lower volume or quantity, showed a rather low prevalence of 10.5%. It is unclear whether this is an independent symptom or a weaker manifestation of the symptom failure to speak. Based on clinical experience, many individuals with SM speak at a lower frequency or in a whisper, suggesting a high prevalence of the symptom in numerous situations. The low prevalence suggests that parents in this study did not distinguish between failure to speak (which was already assumed and should not be reported) and reduced communication, and therefore did not report the latter. For future studies, it would be beneficial to differentiate explicitly between failure to speak and reduced communication and investigate their mutually explanatory variance to determine whether they are distinct or part of the same dimension.

#### Symptoms beyond anxiety

Beyond symptoms that appear to be primarily related to anxiety, parents in this study also reported symptoms that may not be driven by anxiety. Self-esteem impairment/negative affect refers to negative feelings other than anxiety (e.g., helplessness) or reduced self-worth. This symptom is not included in diagnostic criteria for anxiety disorders, but it has been found to be associated with almost all anxiety disorders [25]. In the context of SM, there is evidence that SM is associated with reduced self-esteem. This is supported not only by the current study, but also by the study of Remschmidt et al. [8], in which individuals reported a decrease in their self-esteem. A crucial question in this context is whether reduced self-esteem is a result of not speaking in certain situations or whether it leads to a failure to speak. It should be noted that this category may not necessarily be an inherent symptom of SM, but rather a result of SM that has persisted for some time. A recent study has shown that individuals with (former) SM have lower self-esteem in adulthood than individuals without SM [26]. This may indicate that impaired self-esteem might be a consequence or at least still be present during adulthood. On the other hand, SM typically has a pre-school onset [7], earlier than most other anxiety disorders. Longitudinal studies in children with selective mutism are needed to disentangle the causal relation between selective mutism and reduced self-esteem. Externalizing behaviors may also occur as part of a fear response (e.g. temper tantrums) in children. However, it is possible that the reported symptoms from the externalizing behavior category are not solely related to fear-related situations. This is reasonable because some children with SM do not appear to have elevated levels of anxiety [20] and may present with a diagnosis of oppositional defiant disorder [4]. The reports assigned to this category suggest that a subset of individuals exhibit aggressive behavior that goes beyond tantrums. This subset does not only report anger or temper tantrums but also instances of aggressive behavior (“boxes or beats us parents”). It is important to note that subjective evaluations have been excluded from this analysis. However, due to the method of the study, it is not possible to differentiate which reported symptoms in this category are fear responses and which are classified as fear-independent oppositional or aggressive behaviors. To our knowledge, reduced body tension and slackness has not been previously reported in systematic research on SM yet. It is interesting to note that this symptom appears to be opposing to the symptom of freezing, which is associated with increased body tension. Although reduced body tension was present in only a small subset of children and adolescents with SM in the study at hand, this could indicate a subgroup of individuals with SM characterized by a specific pathomechanism. This highlights the fact that certain phenomena in children and adolescents with SM remain unidentified. It seems necessary to conduct basic psychophysiological research as well as qualitative research to gain a better understanding of this phenomenon in the affected individuals.

In this study, it was found that symptoms, excluding fear, did not show any correlation with SM severity. One possible interpretation is that, apart from the core symptom of failure to speak, only fear is an integral symptom of SM. However, this seems unlikely given the high prevalence of symptoms such as freezing or avoidance. It is more probable that the low correlations are due to the categorical data structure and the associated low variance.

### Clinical and research implications

Considering SM as a mental disorder with a number of additional symptoms beyond failure to speak may have important implications for a better understanding of SM and thus for improving detection and treatment for affected children. Differentiating diagnostic criteria based on empirically identifiable symptoms seems promising in distinguishing SM from normative periods of silence [49] or from social anxiety disorder. Given the heterogeneity of symptoms indicated by the present study, individuals with SM might present with different symptoms and symptom profiles. Modularizing therapy for SM could be a promising approach, as different individuals displaying different symptom profiles may benefit from different interventions. Future studies should focus on subgroups based on empirically identified symptom profiles as these may be related to certain pathomechanisms that require different interventions in the treatment of SM. As we have solely focused on the frequencies of symptoms in individuals with SM, it is crucial to investigate the burden and impairment that these symptoms cause in affected children and adolescents. Moreover, interactions between symptoms should also be addressed as they may offer insights into the maintenance of the disorder (e.g. relation between avoidance and fear).

### Strength and limitations

The current study has both strengths and limitations. One strength is the replication of findings on symptoms of SM from previous studies and the addition of symptoms that have not yet been systematically described in the literature. The study employed a qualitative approach with an open response format, resulting in the first comprehensive overview of possible symptoms of SM without any prior theoretical constraints or specified symptoms. In contrast to most previous studies, we provided a comprehensive description of the possible symptom categories with prototypical examples. The high interrater reliability indicates that symptoms were assigned to intersubjectively reasonable and discriminative symptom categories. However, it should be noted that the survey was conducted online and individuals were included based on a screening instrument rather than a clinical interview. While we were able to conduct a nationwide study and collect a representative sample using a screening instrument that distinguishes well between children with and without SM, we were unable to make a comprehensive diagnosis of SM or identify any comorbidities in our sample. Therefore, it is possible that the symptoms reported in this study may be related to other syndromes besides SM. However, it may not be possible to provide an overview of 'pure' SM symptoms, given the significant symptom overlap typically observed in related syndromes, the prevalence of comorbidities in clinical practice, and the presence of various subclinical symptoms in any individual. Additionally, it is important to note that we only asked about situations in which there was an expectation to speak, so symptoms may be different in other situations (e.g. social situations in which there is no expectation to speak). It is important to note that the survey only included parents' reports of their children's symptoms, rather than the children or adolescents themselves. This may explain why the symptom categories mainly describe observable behavior. The study design does not allow for the assessment of concrete internalizing symptoms from the children's perspective, such as cognitions or fear content. Finally, the study did not take into account factors such as socio-economic status or parental mental disorders. Therefore, it is questionable to what extent the results can be generalised to the entire patient population.

## Conclusion

The study suggests that children and adolescents with SM experience symptoms in addition to failure to speak, suggesting that the current diagnostic criteria for SM in DSM-5 do not fully describe the syndrome of SM. Utilising an open-response format, we have identified a number of different symptoms in individuals with SM, suggesting that SM is more than failure to speak. The presented outline of possible SM symptoms is an important first step for further research into symptoms of SM. These symptoms should be quantitatively investigated using larger samples, particularly with regard to their frequency and relevance (e.g. impairment). Understanding SM as a heterogeneous mental disorder with multiple symptoms and potential subtypes and underlying pathomechanisms has significant implications for its diagnosis and treatment.

## Data Availability

Not available in a public repository at the moment.

## References

[1] American Psychiatric Association (2013) Diagnostic and statistical manual of mental disorders. DSM-5, 5. ed. American Psychiatric Publishing, Washington, DC

[2] Vogel F, Gensthaler A, Stahl J et al (2019) Fears and fear-related cognitions in children with selective mutism. Eur Child Adolesc Psychiatry 28(9):1169–1181. 10.1007/s00787-019-01281-0 30684088 10.1007/s00787-019-01281-0

[3] Muris P, Ollendick TH (2015) Children who are anxious in silence: a review on selective mutism, the new anxiety disorder in DSM-5. Clin Child Fam Psychol Rev 18(2):151–169. 10.1007/s10567-015-0181-y 25724675 10.1007/s10567-015-0181-y

[4] Kearney CA, Rede M (2021) The heterogeneity of selective mutism: a primer for a more refined approach. Front Psychol 12:700745. 10.3389/fpsyg.2021.700745 34177747 10.3389/fpsyg.2021.700745 PMC8222660

[5] Milic MI, Carl T, Rapee RM (2020) Similarities and differences between young children with selective mutism and social anxiety disorder. Behav Res Ther 133:103696. 10.1016/j.brat.2020.103696 32763498 10.1016/j.brat.2020.103696

[6] Schwartz RH, Freedy AS, Sheridan MJ (2006) Selective mutism: are primary care physicians missing the silence? Clin Pediatr 45(1):43–48. 10.1177/000992280604500107 10.1177/000992280604500107 16429215

[7] Viana AG, Beidel DC, Rabian B (2009) Selective mutism: a review and integration of the last 15 years. Clin Psychol Rev 29(1):57–67. 10.1016/j.cpr.2008.09.009 18986742 10.1016/j.cpr.2008.09.009

[8] Remschmidt H, Poller M, Herpertz-Dahlmann B et al (2001) A follow-up study of 45 patients with elective mutism. Eur Arch Psychiatry Clin Neurosci 251(6):284–296. 10.1007/pl00007547 11881843 10.1007/pl00007547

[9] Fried EI (2022) Studying mental health problems as systems. Not Syndromes Curr Dir Psychol Sci 31(6):500–508. 10.1177/09637214221114089

[10] Wilshire CE, Ward T, Clack S (2021) Symptom descriptions in psychopathology: How well are they working for us? Clin Psychol Sci 9(3):323–339. 10.1177/2167702620969215

[11] Ward T, Clack S (2019) From symptoms of psychopathology to the explanation of clinical phenomena. New Ideas Psychol 54:40–49. 10.1016/j.newideapsych.2019.01.004

[12] Ford MA, Sladeczek IE, Carlson J et al (1998) Selective mutism: phenomenological characteristics. Sch Psychol Q 13(3):192–227. 10.1037/h0088982

[13] Vogel F, Gensthaler A, Schwenck C (2022) Frozen with fear attentional mechanisms in children with selective mutism. Cogn Therapy Res. 10.1007/s10608-021-10289-3

[14] Vogel F, Reichert J, Schwenck C (2022) Silence and related symptoms in children and adolescents: a network approach to selective mutism. BMC Psychology 10(1):271. 10.1186/s40359-022-00956-9 36384568 10.1186/s40359-022-00956-9 PMC9670669

[15] Gensthaler A, Dieter J, Raisig S et al (2020) Evaluation of a novel parent-rated scale for selective mutism. Assessment 27(5):1007–1015. 10.1177/1073191118787328 30010386 10.1177/1073191118787328

[16] Fried EI, Epskamp S, Nesse RM et al (2016) What are “good” depression symptoms? Comparing the centrality of DSM and non-DSM symptoms of depression in a network analysis. J Affect Disord 189:314–320. 10.1016/j.jad.2015.09.005 26458184 10.1016/j.jad.2015.09.005

[17] Elo S, Kyngäs H (2008) The qualitative content analysis process. J Adv Nurs 62(1):107–115. 10.1111/j.1365-2648.2007.04569.x 18352969 10.1111/j.1365-2648.2007.04569.x

[18] Revelle W (2024) psych: procedures for psychological, psychometric, and personality research. Northwestern University, Evanston, Illinois. R package version 2.4.3. https://CRAN.R-project.org/package=psych

[19] Christian C, Williams BM, Hunt RA et al (2021) A network investigation of core symptoms and pathways across duration of illness using a comprehensive cognitive-behavioral model of eating-disorder symptoms. Psychol Med 51(5):815–824. 10.1017/S0033291719003817 31907093 10.1017/S0033291719003817

[20] Driessen J, Blom JD, Muris P et al (2020) Anxiety in children with selective mutism: a meta-analysis. Child Psychiatry Hum Dev 51(2):330–341. 10.1007/s10578-019-00933-1 31650460 10.1007/s10578-019-00933-1 PMC7067754

[21] Hofmann SG, Hay AC (2018) Rethinking avoidance: toward a balanced approach to avoidance in treating anxiety disorders. J Anxiety Disord 55:14–21. 10.1016/j.janxdis.2018.03.004 29550689 10.1016/j.janxdis.2018.03.004 PMC5879019

[22] Mireault G, Trahan J (2007) Tantrums and anxiety in early childhood: a pilot study. Early Childhood Res Pract 9(2):8

[23] Steimer T (2002) The biology of fear- and anxiety-related behaviors. Dialogues Clin Neurosci 4(3):231–249. 10.31887/DCNS.2002.4.3/tsteimer 22033741 10.31887/DCNS.2002.4.3/tsteimer PMC3181681

[24] Troisi A (2002) Displacement activities as a behavioral measure of stress in nonhuman primates and human subjects. Stress 5(1):47–54. 10.1080/102538902900012378 12171766 10.1080/102538902900012378

[25] Maldonado L, Huang Y, Chen R et al (2013) Impact of early adolescent anxiety disorders on self-esteem development from adolescence to young adulthood. J Adolesc Health 53(2):287–292. 10.1016/j.jadohealth.2013.02.025 23648133 10.1016/j.jadohealth.2013.02.025 PMC3725205

[26] Tomohisa Y, Yumi I, Inoue M (2022) Long-term outcome of selective mutism: factors influencing the feeling of being cured. Eur Child Adolesc Psychiatry. 10.1007/s00787-022-02055-x 35984502 10.1007/s00787-022-02055-x

